# Could AMPs and B-cells be the missing link in understanding periodontitis?

**DOI:** 10.3389/fimmu.2022.887147

**Published:** 2022-09-21

**Authors:** Vanessa Dominique Lobognon, Jean-Eric Alard

**Affiliations:** ^1^ B lymphocytes, Autoimmunity and Immunotherapies (LBAI), Mixed Research Unit (UMR)1227 INSERM, University of Brest, Brest, France; ^2^ Service d’Odontologie, University Hospital (CHU) de Brest, Brest, France

**Keywords:** B cell, anti-microbial peptides, periodontal diseases, periodontitis, immunomodulation

## Abstract

Periodontal diseases are common inflammatory conditions characterized by bone loss in response to simultaneous bacterial aggression and host defenses. The etiology of such diseases is still not completely understood, however. It has been shown that specific pathogens involved in the build-up of dysbiotic biofilms participate actively in the establishment of periodontitis. This multifactorial pathology also depends on environmental factors and host characteristics, especially defenses. The immune response to the pathogens seems to be critical in preventing the disease from starting but also contributes to tissue damage. It is known that small molecules known as antimicrobial peptides (AMPs) are key actors in the innate immune response. They not only target microbes, but also act as immuno-modulators. They can help to recruit or activate cells such as neutrophils, monocytes, dendritic cells, or lymphocytes. AMPs have already been described in the periodontium, and their expression seems to be connected to disease activity. Alpha and beta defensins and LL37 are the AMPs most frequently linked to periodontitis. Additionally, leukocyte infiltrates, especially B-cells, have also been linked to the severity of periodontitis. Indeed, the particular subpopulations of B-cells in these infiltrates have been linked to inflammation and bone resorption. A link between B-cells and AMP could be relevant to understanding B-cells’ action. Some AMP receptors, such as chemokines receptors, toll-like receptors, or purinergic receptors, have been shown to be expressed by B-cells. Consequently, the action of AMPs on B—cell subpopulations could participate to B-cell recruitment, their differentiation, and their implication in both periodontal defense and destruction.

## Introduction

Periodontal diseases are inflammatory conditions with an infectious etiology that can involve various pathogens such as *Aggregatibacter actinomycetemcomitans*, *Fusobacterium nucleatum*, *Porphyromonas gingivalis*, *Prevotella intermedia*, *Treponema denticola*, and *Treponema forsythia* (1). Periodontal diseases are the most common oral diseases in the world, with the moderate form affecting 45–50% of adults and the severe form 9–11% (2, 3). They can be divided into two main types: gingivitis and periodontitis. Periodontitis are chronic, multifactorial, immuno-inflammatory diseases associated with dysbiotic bacterial biofilms. They are characterized by the progressive destruction of the supporting apparatus of the teeth, often leading to tooth loss and finally to bone resorption (4, 5). The appearance and evolution of these diseases are dependent not only on the pathogenicity of periodonto-pathogenic bacteria, but also on genetics and environmental and behavioral risk factors that affect host susceptibility (1). In addition to being a worldwide public health concern, these diseases provide a strategic model to study inflammatory processes. The oral localization of the periodontium provides access for both clinical assessment and tissue biopsy, facilitating longitudinal studies. Furthermore, the mouth is the first place for most interactions between the self and non-self and is involved in immune system training from birth with probable heritability of microbiota (6). Any advances in the understanding of periodontal disease etiopathogenesis could improve the overall knowledge of chronic inflammation and some systemic conditions such as cardio-metabolic, neurodegenerative, and autoimmune diseases and cancer (1).

In the oral cavity, when facing periodontal pathogens, the innate and adaptive immune systems cooperate to combat this bacterial attack (7). One mechanism of the innate immune system to fight oral infections is the release of antimicrobial peptides (AMPs) (8–10). This appellation gathers small molecules (between 10 and 60 amino acids) expressed in animals with, most of the time, cationic properties (11). Produced by many cells, including neutrophils, macrophages, dendritic cells, and even lymphocytes, AMPs play an important role in innate immunity due to their rapid and broad-spectrum antimicrobial activity (12–15). These molecules are capable of neutralizing a large number of pathogenic microorganisms, including bacteria, fungi, parasites, and viruses (16–18). They have also been shown to perform various biological activities within the innate and adaptive immune systems (18–23). They can induce both chemoattraction and/or cell activation, and they also play a role in inflammation resolution (20). Their known targets are mainly neutrophils, monocytes, dendritic cells, and, to a lesser extent, T cells (24). They can also work in synergy with the cell mediator’s cytokine and chemokine, as has been shown for HNP1and RANTES (25) or IL1β (26).

AMPs have also been suggested to potentiate the innate immune response and function as a bridge between innate and adaptive immunity by regulating B and T lymphocytes and natural killer cells (27–29). Indeed, an increasing number of studies have been conducted on the action of AMPs in the regulation of T-cells (30, 31) mainly with TH17 response linked to cathelicidins, but few have explored B-cells, more precisely plasma cells (32). Until recently, the immunomodulatory functions of AMPs have been poorly studied for periodontitis, as most of the approaches were focused on their antimicrobial roles. The first therapeutical approach to these diseases was to target pathogenic bacteria, and AMPs are promising candidates for this purpose (33). However, managing the host reaction seems to be crucial to reach a potential curative strategy, as the appearance of the disease cannot be reduced to only a bacterial presence (34). Furthermore, AMPs are in the frontier between these two strategies, as they can act on both bacteria and leucocytes. Recent research on the immune response during periodontitis has underlined the role of B-cells in both defense mechanisms and bone resorption (35). Few connections, however, have been reported between B-cells and AMPs. This blind spot could be a critical handicap in the understanding of periodontal disease etiology and, more widely, chronic inflammatory diseases. The objective of this review is to answer to the pertinence of studying AMP action of B-cells. In this purpose, it is first needed to sum up which peptides are relevant in periodontal disease, what impact B-cells have on periodontitis, and if they can be a target for AMP. Peptides sharing both a role in periodontitis and a potential receptor on B-cells would be promising candidates for further analysis.

## Functions of antimicrobial peptides in periodontal diseases

Oral infections are first managed by the innate immune system, especially with AMPs. Salivary gland epithelial cells, neutrophils, monocytes, and potentially other cells secrete these AMPs in the oral cavity. Their antimicrobial activity against oral pathogenic bacteria and their biofilms plays a central role in oral microbiota homeostasis (33). Three major families of AMP are found in saliva: defensins, cathelicidins, and histatins (34, 36). AMPs are primarily known for their antibacterial activity (elimination or inhibition of the growth of these microorganisms). In addition, they show antifungal and/or antiviral effects against a large number of other microorganisms (37, 38) and even exhibit antitumoral properties (39). Theses lytic activities are linked to AMPs’ capacity to create pores in their targets.

Independently of their antimicrobial activities, AMPs may play a role in the inflammatory response and the immune response, even serving as a link between the innate and adaptive immune responses (35). Their antimicrobial effect and their role in the regulation of immune responses are two aspects of particular interest in relation to periodontal disease. Natural AMPs have shown antibacterial effects against periodontal pathogens. Healthy subjects show distinct levels of natural AMPs compared to those with periodontitis (40), with most studies reporting higher levels of LL37, beta defensins, or HNP1, although the result remains heterogeneous between studies. The most studied AMPs in relation to the periodontal state are LL-37 and alpha and beta defensins (41).

In the literature, several roles have been reported for AMPs in periodontal disease, such as inhibiting the growth of pathogenic bacteria (LL37, HBD-1, HBD-2, HBD-3, histatin-2, HNP1, HNP2, HNP3), promoting periodontal tissue healing (LL37, HBD-1, HBD-2, HBD-3, histatin-2), promoting bone healing (HBD-2, histatin-1), and serving as a potential indicator of the severity of periodontal disease (HBD-2, HNP1, HNP2, HNP3). Additionally, AMPs have been reported to promote osteogenic differentiation and reduce bone loss (42). Histatin-5 has been shown to be an inhibitor of host and bacterial enzymes involved in periodontal destruction (43). Among natural AMPs, HBD are expressed in the buccal bone (44). HBD have been shown to promote the proliferation of human mesenchymal stem cells, osteoblasts, and keratinocytes in cultures (45). In addition, HBD and histatin-1 promote bone regeneration and prevent infection (46–48).

All these findings suggest that the AMPs relevant to the management of periodontal diseases could be LL-37, alpha and beta defensins, and histatins. In contexts other than the oral cavity immunity, these AMPs have all been described as immune modulators. Alpha defensins have been shown to inhibit monocyte/macrophage-produced pro-inflammatory cytokines such as IL6 and IL1β and to increase leucocyte recruitment directly or indirectly. Beta defensins can either be decreased or increased (49). Similar chemoattraction has been reported for beta defensins and LL37 with additional impact on immune cells’ signaling pathways (49). LL37 is strongly connected to neutrophil extracellular traps (NETs): DNA strands, released by dying neutrophils, that are covered by multiple proteins and peptides that play critical roles during both inflammation and microbial neutralization (50). These NETs activate BAFF production, which is a major B-cell activator (50). This activation could be critical, as neutrophils are strongly connected to periodontal diseases, and B-cells have been shown to be effectors of a part of periodontitis pathogenesis.

## Functions of B-cells in periodontal diseases

The ontogenesis of B-cells takes place in the bone marrow from pluripotent hematopoietic stem cells (51). The latter form lymphocyte progenitors, some of which migrate to the thymus for the formation of T cells, while the rest remain in the bone marrow to form B-cells until the immature B stage. During antigen-independent maturation in the marrow, there are four distinct stages: the early pro-B cell, the late pro-B cell, the pre-B cell (precursor B), and the immature B cell. Immature B-cells then migrate to secondary lymphocyte organs (spleen, lymph nodes, tonsils, and lymphoid tissues associated with mucous membranes) in the form of transitional B-cells (52). These transitional B-cells transform into either marginal zone cells or follicular cells. All of these cells undergo a succession of T-independent or T-dependent differentiation, leading to the formation of memory cells or plasma cells (52).

Few studies have highlighted the roles of B-cells in periodontitis. Certain roles of B-cells in periodontitis have been recently discovered, however. A review of the literature showed the role of B-cells in periodontitis and the potential interest in using B-cells as a target for new treatments for severe periodontitis (53). Additionally, a study carried out by Demoersman highlighted the crucial role of B-cells in periodontal disease with an increase of memory B-cells and a reduction of their regulatory counterpart in severe forms (54). It has also been shown that anti-B lymphocyte therapy could be beneficial in improving periodontitis, suggesting a major role of B-cells in this disease (55). The 2015 study by Abe et al. 2015 suggests that B-cells have a more important role than T cells in bone resorption (56). It has been reported that, in periodontitis, the presence of B-cells specific to periodontal pathogenic bacteria is essential for the establishment of the bone resorption characteristic of periodontitis (57). Some authors have also suggested an involvement of B-cells in bone remodeling (58–60).

Bacterial infection remains important in the etiopathogenesis of periodontal disease. An immuno-inflammatory response is set up by the body against this bacterial infection. This reaction is the origin of the destruction of supporting tissues of the tooth, loss of attachment, and alveolar bone lysis. Inflammation-induced osteoclast genesis in periodontal disease involves several pathways of mechanisms that involve several biological molecules and their products. Cells of the B lymphoid lineage can contribute to the physiopathology of bone disorders by regulating osteoclast genesis in the context of periodontal infection through several pathways of mechanisms. The molecular mechanism pathways by which cells of the B lymphoid lineage regulate osteoclast genesis in periodontal disease have become better understood in recent years. As well as their antibody secretion, B-cells contribute to the destruction of the alveolar bone in RANKL-dependent periodontitis (61). B-cells express RANKL, a protein involved in osteoclast differentiation, activation, and survival. RANKL then binds to its RANK receptor expressed by osteoclast precursor cells and preosteoclasts to stimulate their differentiation into osteoclasts resorbing alveolar bone (59, 62–65). In periodontitis, the main source of RANKL are T-cells and B-cells (66), either of which themselves serve as progenitors of osteoclasts. Normal pro-B-cells may serve as osteoclast progenitor cells (67).

B-cells express SOFAT, an osteoclastogenic cytokine independent of RANKL. By stimulating osteoblasts, SOFAT modulates the production of osteoclastogenic cytokines and contributes to osteoclast formation and bone destruction in periodontitis. Human B-cells, plasma cells, and T-cells express SOFAT, which is a bone-destroying factor in periodontal disease lesions (68–70).

Pathogens that breach periodontal tissue express molecular patterns associated with pathogenic agents (PAMPs) such as lipopolysaccharides, lipoteichoic acid, peptidoglycan, bacterial DNA, and double-stranded RNA (71, 72). These PAMPs can be recognized by receptors such as Toll-like receptors **(**TLR) expressed on immune cells: macrophages, Langerhans cells, dendritic cells, and polymorphonuclear neutrophils. They can also be recognized by epithelial cells, gingival fibroblasts, fibroblasts of the periodontal ligament, osteoblasts, osteoclasts endothelial cells, and even lymphocytes (73, 74). The interaction between macrophages, dendritic cells, neutrophils, and PAMPs *via* TLRs leads to the production of pro-inflammatory cytokines and chemokines such as TNF-α, IL-1, IL-6 (CXCL-8: IL-8), IL-12, and IL-18. It should be noted, however, that dendritic cells act as antigen-presenting cells for B and T cells (19, 75–77). Neutrophils are one of the first inflammatory cells to arrive at the site of periodontal inflammation by chemotaxis (78), following chemoattractant such as IL-8 secreted by oral epithelial cells, connective fibroblasts, and immune cells (79) and growth-related gene product-α (80). Neutrophils contain cytoplasmic granules that in turn contain lytic enzymes and molecules with antimicrobial properties such as cathepsins, lactoferrin, lysozyme, and defensins, which destroy microorganisms (81).

Defensins are AMPs known for their antibacterial activity (elimination or total inhibition of the growth of these microorganisms), and several roles have been attributed to them in relation to periodontal diseases, such as inhibiting the growth of pathogenic bacteria, promoting the healing of periodontal tissues, promoting bone healing, and serving as a potential indicator of the severity of periodontal disease (42). TNF-α, IL-1, and IL-6 are osteotropic cytokines that stimulate osteoclast resorption in periodontitis (82) and are found in higher concentrations in patients with periodontal disease than in healthy individuals (83).

**Figure 1:**
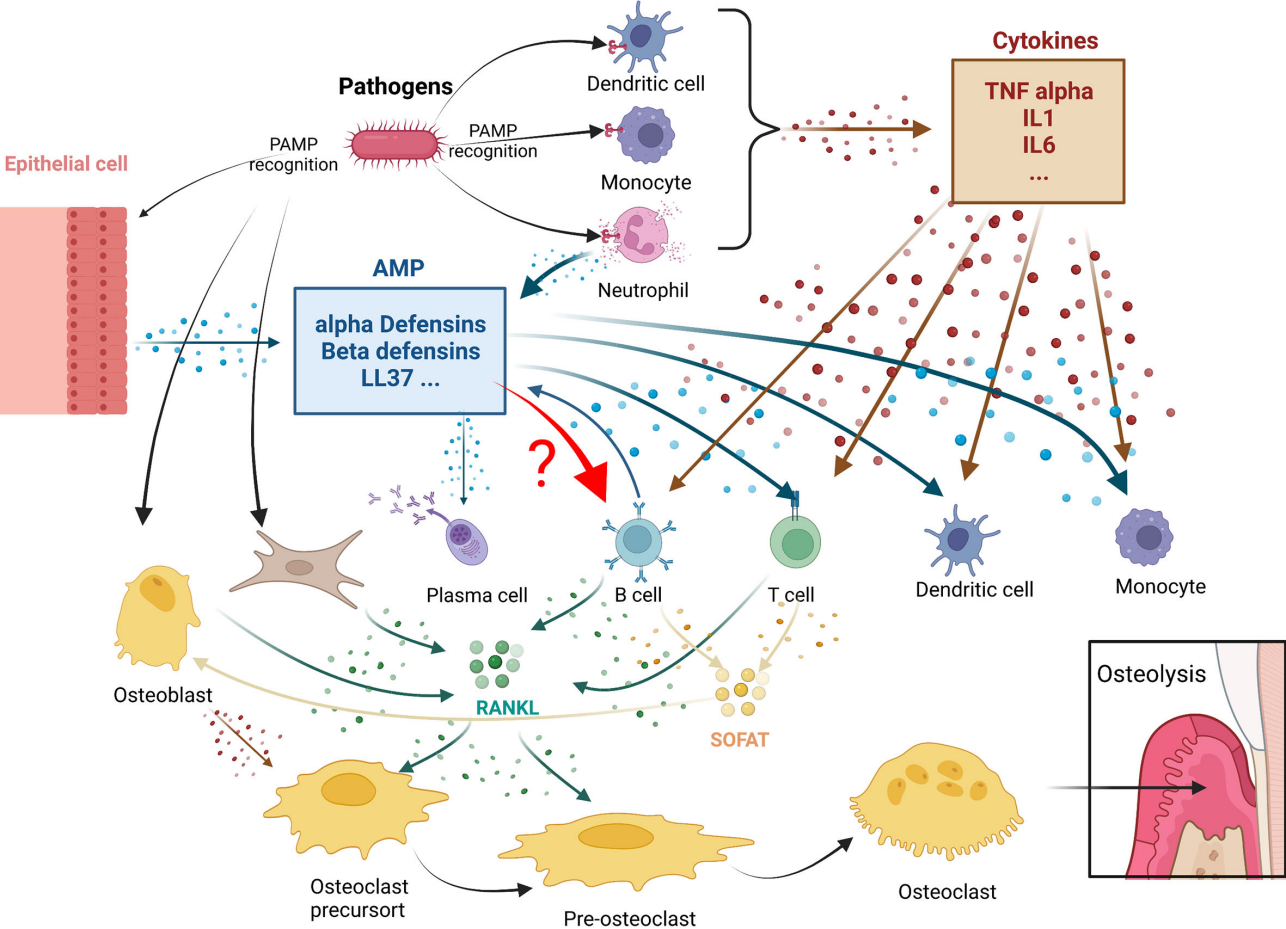
Antimicrobial peptides immunomodulation in periodontitis osteolysis: potential connections with leukocytes. In periodontitis, pathogens triggers both cytokines (in brown) or antimicrobial peptides (AMPS) (in blue) production. Both mediator’s categories are known to play roles in leukocytes actions. A crucial step in these diseases is the expression of RANKL and SOFAT. B and T cells, which are the main source of RANKL and SOFAT, are central in this orchestration of osteoclast differentiation and activity leading to osteolysis. The effect of AMPs in B in this environment still remain to be determined.

Cells of the B lymphoid lineage can participate in osteoclast genesis through two main pathways (**Figure 1**):

1. The B-cells express RANKL, the main factor involved in the differentiation, activation, and survival of osteoclasts. TNF-α secreted by macrophages and dendritic cells can stimulate osteoblasts, T cells, and B-cells to produce RANKL. In addition, periodontal ligament fibroblasts and gingival fibroblasts can regulate osteoclast activity by secreting RANKL (58, 84).

2. The B-cells express SOFAT, which is a bone-destroying factor in periodontal disease lesions (70), independent of RANKL. By co-opting osteoblasts to increase production of osteoclastogenic cytokines, SOFAT can exacerbate inflammation and promote osteoclast formation and bone destruction (68).

In all these interactions that occur during periodontal disease, however, it is not known whether there is a direct relation between AMPs and B-cells.

## Can antimicrobial peptides affect the function of B-cells in periodontal diseases?

**Figure 2:**
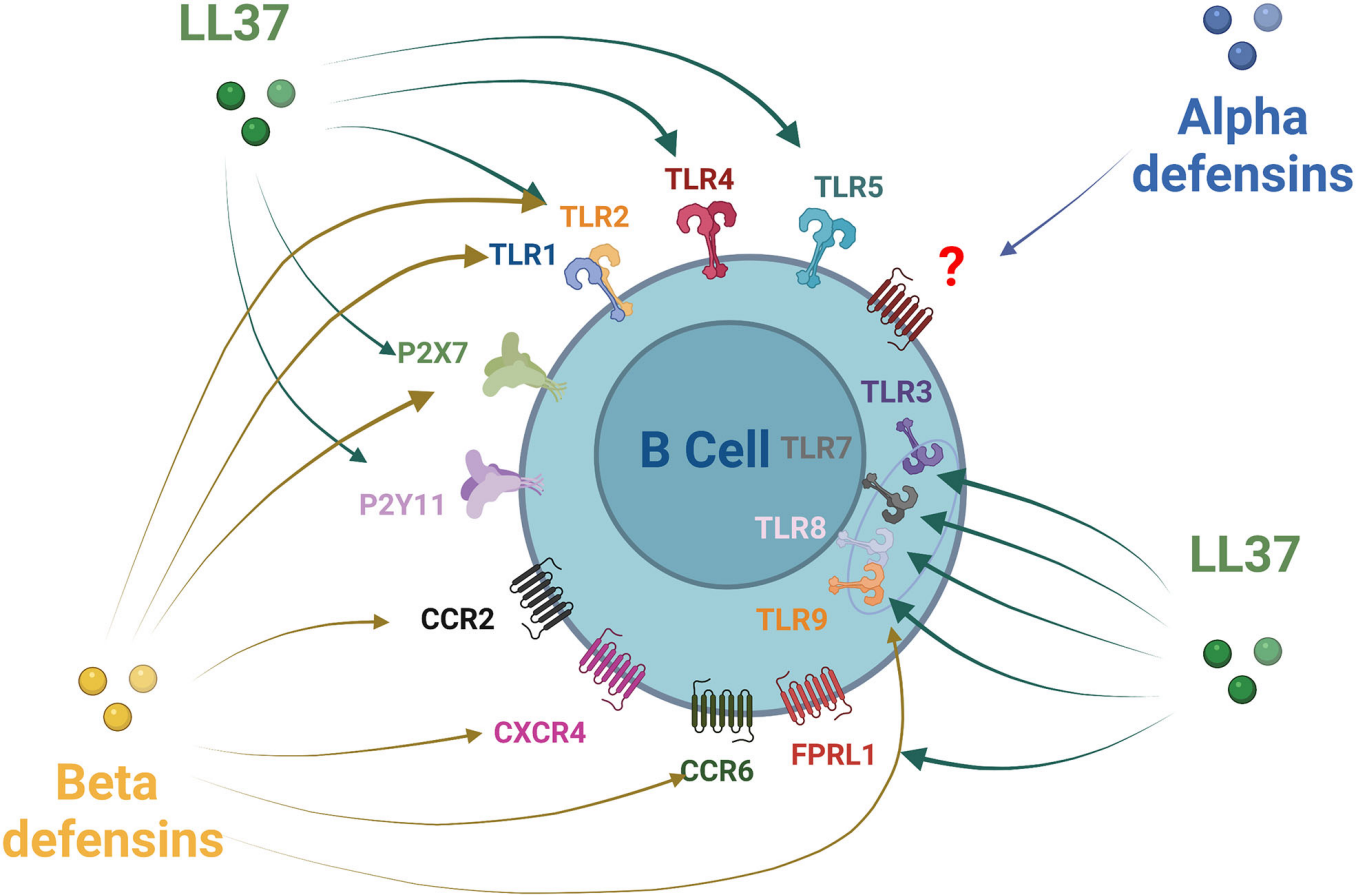
Antimicrobial peptides receptors expression on B-cells. Alpha defensin has been shown to recruit B-cells through a non-identified Protein G Coupled Receptor. Beta defensin family receptors are more documented with an action of HBD1 and HBD2 on CCR6 and HBD3 *via* CCR6, CCR2, CXCR4, P2X7, TLR1, TLR2, and TLR9. LL37 act on a larger number of receptors that can be expressed by B-cells: TLRs1-5, TLRs7-9, FPRL1 P2X7, and P2Y11.

Most of the effects of AMPs on host cells are mediated *via* the specific activation of various cell surface receptors, membrane channels, or specific intracellular targets and pathways (85, 86). Relations have been established between the LL-37 peptide and at least nine receptors of different classes, including four G-protein-coupled receptors, three receptor tyrosine kinases, a ligand-gated ion channel, and TLRs (87, 88) (**Figure 2**). Several receptors and pathways involved in the immune functions of human beta defensins have been studied, such as TLRs, receptors of the purinoceptor family, and chemokine receptors (85) (**Figure 2**). Some receptors have been described for beta defensin, such as CCR6, CCR4, CCR2, TLR1/2/9, and P2X7 (85). However, specific alpha-defensin (HNP) receptors have not yet been clearly identified, only a G-protein-coupled receptor response has been confirmed so far (85). B-cell expression of some of these receptors conditioned their responsiveness to AMPs. Many chemokine receptors regarded as AMP receptors are expressed on T cells but can also be found on human B-cells, such as CXCR4 (89), CXCR5, CXCR3, CCR7, CCR1 (90, 91), CCR2 (92, 93), and CCR6 (94, 95). CCR6 probably plays an important role in B-cell trafficking in humans and is established as an efficient receptor on human B-cells (92). B-cells express several TLRs. TLR1, TLR2, TLR4, TLR5, TLR6, and TLR10 receptors are expressed on the cell membrane, while TLR3, TLR7, TLR8, and TLR9 are expressed in the endosomes. Some of them can identify AMPs such as TLR1, TLR2, and TLR9 (95–100). Their signaling in B-cells is related to the stage of activation and the tissue situation of the B-cells (101). It has also been reported that certain receptors of the purinoceptor family, such as P2X7 and P2Y11, are expressed on B-cells (102, 103).

Furthermore, the literature also mentions that B-cells themselves express AMPs such as alpha defensins (HNP-1–3), HBD-2, and cathelicidin LL-37 in the presence of pathogens such as *Aggregatibacter actinomycetemcomitans* (104, 105). Thus, B-cells can produce some of the AMPs found in periodontal tissue and potentially react to them. For now, however, the only clear link between B-cell lineage and their response to AMPs is the IgG production induced in plasma cells by LL-37 associated with NETs during systemic lupus erythematosus (32). If this IgG production is useful to predict the installation of the disease and participate in host defense. initially, no direct connection to osteolysis, the major concern in periodontitis, was described (106). More recently, anti-carbamylated LL37 autoantibodies have been linked to an increase in bone resorption in RA (107). Auto-immunity and periodontitis are connected in many ways. Patients with auto-immune conditions such as Lupus or RA are known to be more prone to developing periodontitis (55, 108). Bone destruction in periodontitis shares similarities with tissue damage observed during RA and presents a TH17 cytokines overexpression that can be also found in RA, Lupus psoriasis, and many other auto-immune conditions (109, 110). In addition, AMPs’ participation in autoimmunity has been investigated in the past few years. High concentrations of AMPs have been found in sera of Lupus patients or the joints of RA patients (107, 111, 112). For now, AMPs’ immunomodulatory role in autoimmunity is not fully understood, probably due to the difficulty in overcoming AMPs/cytokines/chemokines’ roles redundancy combined with dual detrimental and protective effects (113, 114). Regardless, the role of autoantibodies against AMPs can be critical in autoimmunity. LL37/DNA complexes during NETs release can be carbamylated or citrullinated during autoimmune disease, which can be the result of oral cavities pathogens such as PG or AA (115, 116). Modified LL37s can then be recognized by specific autoantibodies leading to the formation of immune complexes that have been described in Lupus, auto-immunes vasculitis, psoriasis, and RA (117–119). The fact that in RA such autoantibodies participate in osteolysis strengthens the idea of the implication of AMP in autoimmunity but also in inflammatory contexts such as periodontitis.

All these elements point to an implication of B-cells by their production of autoantibodies against AMPs in autoimmunity with, in parallel, a potential immunomodulatory action that remains to be investigated. For now, however, no causal relationship has been established between AMPs’ immunomodulation and B-cells participation in osteolysis. Presence of autoantibodies against AMPs could be of interest as an actor of osteolysis and potentially as a biomarker in diseases associated with bone self-destruction. As previously mentioned, a large number of AMPs have been identified in the oral cavity, and several exhibit antimicrobial effects against periodontal pathogens. They offer a broad spectrum of roles (antibacterial, antiviral, and/or antifungal or immunomodulation activities) that are critical in periodontal diseases. These characteristics make these natural molecules promising candidates for anti-infection strategies. Moreover, it has been proposed that the differential regulation of AMPs in periodontal disease makes them relevant biomarkers for the disease in saliva and gingival fluid (41). Promising strategies in the treatment of periodontitis can be derived from AMPs in periodontal diseases. They could be considered as a potential alternative to traditional antimicrobial therapy (antibiotics) in periodontal infections (38). Nevertheless, their immunomodulatory action must be better understood to avoid any unwanted effect. In this regard, B-cells’ involvement in AMP immunomodulation also needs to be known.

## Conclusion

As only sporadic information is available on the effect of AMPs on B-cells, it may be tempting to think that they have no or few connections. However, the B lymphocyte family consists of various subtypes with specific patterns of receptors and functions. Global analysis of lymphocytes tends to be disconnected from functional reality, and the results are unclear due to the heterogeneity and low number of each category of cell. The only way to overcome this limitation is to study each lymphocyte subpopulation. This, however, has not been done for the immunomodulatory action of AMPs on B-cells. Only plasma cells and LL37 have been functionally connected. An in vivo or ex vivo tissue analysis would be convenient to assess this link due to the small B-cell subpopulation. In addition, AMPs have been shown to be critical in both the initiation and the chronicity of inflammation. As AMPs are already known to influence and participate actively in both the initiation and the persistence of inflammation (120), it would be unlikely that they are not involved in periodontitis-related immune system dysregulation. Recent studies on pathogenesis tend to involve more and more B-cells in osteolysis processes. An interaction between B-cells and AMPs could explain the pathogenic action of B-cells. As they can carry some identified AMP receptors, they should react to their presence.

A parallel is frequently made between periodontitis and auto-immune diseases.

In addition, periodontal diseases are chronic inflammatory conditions with changing inflammatory states (1). Longitudinal studies are still rare but may be needed to truly understand the molecular mechanisms. This time-dependent disease activity could explain why studies on AMP presence in periodontitis are frequently contradictory, with AMPs such as LL37 or defensins linked to both protective and pathogenic patterns of the disease. In a similar way, the action of B-cells depends on the result of each subpopulation action found at the same time in tissue. Regulatory B-cells seem decreased or inefficient in situations involving chronic inflammation, which has been confirmed in periodontitis (54). The variability of B-cell subpopulations seems to share some patterns with AMP presence. Elucidation of the relationship between AMPs and B-cells could clarify why the immune system participates in periodontal tissue destruction during its struggle against pathogens, and may clarified whether or not periodontitis can be considered as an autoimmune-like condition. Furthermore, this understanding could open new possibilities for treatments involving the targeting of B-cells and/or AMPs to optimize the immune response and reduce self-damage.

## Data availability statement

The original contributions presented in the study are included in the article/supplementary material. Further inquiries can be directed to the corresponding author.

## Author contributions

All authors contributed to the article and approved the submitted version.

## Conflict of interest

The authors declare that the research was conducted in the absence of any commercial or financial relationships that could be construed as a potential conflict of interest.

## Publisher’s note

All claims expressed in this article are solely those of the authors and do not necessarily represent those of their affiliated organizations, or those of the publisher, the editors and the reviewers. Any product that may be evaluated in this article, or claim that may be made by its manufacturer, is not guaranteed or endorsed by the publisher.
